# Stroke Investigation Group in North and Central London (SIGNAL): cohort profile of a prospective large-scale comprehensive stroke registry

**DOI:** 10.1136/bmjopen-2025-110772

**Published:** 2026-01-30

**Authors:** Hatice Ozkan, Gareth Ambler, Philip S Nash, Simone Browning, Raafiah Mussa, Alex Leff, Hans R Jäger, Parashkev Nachev, Richard Perry, Edgar Chan, Robert Simister, David J Werring

**Affiliations:** 1 UCL Stroke Research Centre, Department of Translational Neuroscience and Stroke, UCL Queen Square Institute of Neurology, London, UK; 2 Comprehensive Stroke Services, National Hospital for Neurology and Neurosurgery, London, UK; 3 Statistical Science, University College London, London, UK; 4 National Hospital for Neurology and Neurosurgery, London, UK

**Keywords:** Stroke, REGISTRIES, Patient Reported Outcome Measures, Brain Injuries

## Abstract

**Abstract:**

**Purpose:**

Large-scale stroke registries can provide critical insights into disease mechanisms, progression and healthcare needs, informing prevention and care. However, few collect detailed demographic, brain imaging, and comprehensive long-term follow-up data. To address this, we established the prospective Stroke Investigation Group in North And central London (SIGNAL) registry in 2017.

**Participants:**

The SIGNAL registry included 3931 adults aged ≥18 years with confirmed acute stroke (cerebral ischaemia or intracerebral haemorrhage (ICH)) admitted to the University College London Hospital hyperacute stroke unit between January 2017 and 2020, drawn from an ethnically diverse North and Central London population (~1.6 million). Baseline data included demographic, clinical, brain imaging and next-of-kin information. Six month follow-up included measures of functional status and non-motor outcomes (anxiety, depression, fatigue, sleep, pain, language, continence, social participation, cognition) via face-to-face, telephone or postal follow-up methods.

**Findings to date:**

The mean age of individuals included in the SIGNAL registry was 72.1 years, and 1806 (45.9%) were female. The ethnic distribution comprised 2365 (60%) white, 649 (16.5%) black and 511 (13%) Asian. Stroke diagnoses included 3371 (85.8%) with cerebral ischaemia and 560 (14.2%) with ICH. On admission, 2240 individuals (57.0%) had a National Institutes of Health Stroke Scale score >4, indicating moderate stroke severity. At hospital discharge, the median functional outcome, measured by the modified Rankin Scale, was 3 (IQR 1–4), indicating moderate disability. At 6 months, functional outcomes measured with mRS were available for 3755 individuals (95.6%) with a median score of 1 (IQR=0–3) and non-motor outcomes were available for 3080 individuals (92.3%). The most prevalent adverse non-motor outcomes were fatigue 1756 (57%), reduced social participation 1694 (55%) and sleep disturbance 1663 (54%).

**Future plans:**

Further analyses of SIGNAL registry data will investigating associations between stroke mechanisms, subtypes and neuroimaging features and 6-month functional status, non-motor outcomes and cognitive impairment. Longer term follow-up of survivors for ~10 years is also planned.

**STRENGTHS AND LIMITATIONS OF THIS STUDY**

- This large, hospital-based prospective stroke registry included 3931 individuals with acute stroke from an ethnically diverse urban population.
- The registry systematically captured detailed clinical, demographic and high-quality brain imaging data within 3 days of stroke onset.
- Comprehensive 6-month follow-up included both functional and multi-domain non-motor outcomes, such as mood, cognition, fatigue, sleep, pain, language, social participation, bowel and bladder dysfunction.
- Multiple overlapping follow-up methods were employed to ensure high retention and minimise differential loss to follow-up.
- As a single-centre registry, findings may not fully represent stroke care and outcomes in other geographic or healthcare settings.

## Introduction

 Stroke is the second leading cause of death worldwide and a major cause of adult disability.^1^ Despite considerable advancements in our understanding of stroke mechanisms and prognosis, particularly through large stroke registries, few studies have integrated detailed stroke subtyping in unselected populations.^2 3^ In the UK, notable contributions to this field include the South London Stroke Register, established in 1995, and the Oxford Vascular Study, initiated in 2002, both of which have provided valuable insights into acute stroke care, cognitive function, prevention, medication management and imaging profiles.^2 3^

A key gap remains in combining standardised stroke classification, incorporating detailed brain imaging, with a full spectrum of functional and non-motor outcomes, particularly across multiple domains in ethnically diverse stroke populations.^1^ Although previous stroke registries have made significant strides in clinical and prognostic research, they have often lacked the depth needed to capture the complexity of patient-reported non-motor outcomes (ie, anxiety, depression, fatigue, sleep disturbance, social participation, pain, bowel dysfunction, bladder dysfunction, mood problems, communication problems, activities of daily living (ADL), memory and thinking problems) across diverse demographic groups and stroke types.^2^,^4^ This is particularly important given the growing recognition of the complex interplay between stroke subtypes, socioeconomic factors, healthcare access and functional and non-motor outcomes with relevance to stroke rehabilitation goals and quality of life after stroke.^5 6^

In 2017, we commenced the routine inclusion of all individuals diagnosed with acute cerebral ischaemia or spontaneous intracerebral haemorrhage (ICH) into the Stroke Investigation Group in North and central London (SIGNAL) registry. SIGNAL is a large-scale, prospective, hospital-based observational stroke registry supported by the University College London Hospitals (UCLH) NHS Foundation Trust’s Biomedical Research Centre (BRC). We included all individuals admitted with a diagnosis of stroke due to acute cerebral ischaemia or spontaneous ICH, who were residents of a North Central London (NCL) population of ~1.6 million, one of five London Integrated Care Systems.^7^

The SIGNAL registry was established to characterise complex clinical phenotypes, evaluate stroke recovery trajectories, investigate underlying mechanisms and capture under-recognised non-motor outcomes to support person-centred care. SIGNAL systematically collected demographic, clinical and brain imaging data at baseline during admission to the comprehensive stroke services at UCLH, with structured 6-month follow-up assessing functional status, recurrent stroke, cognition, language and multi-domain non-motor outcomes (anxiety, depression, fatigue, sleep disturbance, social participation, pain, bowel dysfunction, bladder dysfunction, mood problems, communication problems, ADL). Long-term follow-up for up to 10 years after stroke is planned, subject to access to the necessary funding support.

SIGNAL aims to provide a comprehensive, well-characterised and ethnically diverse database that enables real-world evaluation of prevalence, predictors and underlying mechanisms of complex and under-recognised stroke outcomes, supporting person-centred, data-driven improvements in stroke care.

## Cohort description

### Registry design and participants

SIGNAL is a hospital-based stroke registry that included consecutive individuals residing in one of the five NCL boroughs, including Barnet, Camden, Enfield, Haringey and Islington, who presented to the Comprehensive Stroke Service at UCLH with clinically and radiologically confirmed acute ischaemic stroke or ICH between 1 January 2017 and 13 January 2020. Inclusion of new patients into the registry finished in January 2020 due to the expiry of funding support and effects of the COVID-19 pandemic.

**Figure 1:**
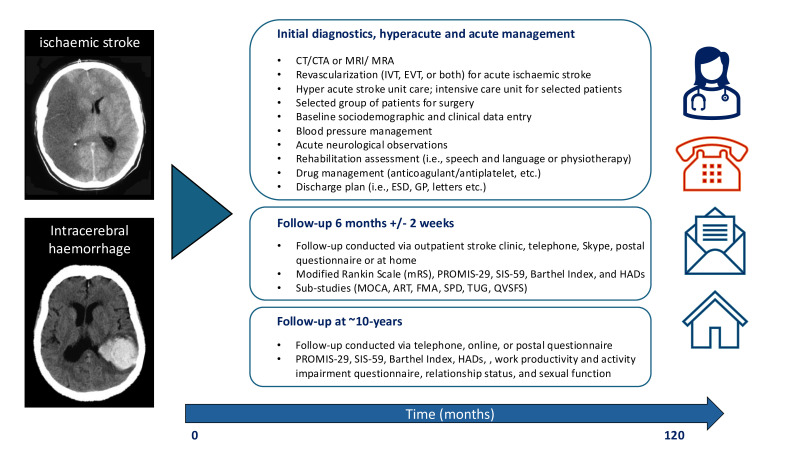
Graphical illustration of SIGNAL registry. *Illustration of data capture and follow-up structure of SIGNAL. ART, Action Research Arm Test; CTA, CT angiography; ESD, early supported discharge; EVT, endovascular thrombectomy; FMA, Fugl-Meyer Assessment; GP, general practitioner; HADS, Hospital Anxiety and Depression Scale; IVT, intravenous thrombolysis; MoCA, Montreal Cognitive Assessment; MRA, magnetic resonance angiography; PROMIS-29, Patient-Reported Outcomes Measurement Information System 29-Item Profile; QVSFS: Questionnaire for Verifying Stroke-Free Status; SIGNAL, Stroke Investigation Group in North and Central London; SIS-59, Stroke Impact Scale 59-Item Version; SPD, Self-Perceived Disability; TUG, Timed-Up-and-Go Test.

The registry captured detailed sociodemographic, clinical and brain imaging data at baseline during admission to the Comprehensive Stroke Service at UCLH, with structured 6-month follow-up to assess functional outcomes, recurrent stroke, cognition, language and multi-domain non-motor symptoms. Comprehensive long-term follow-up data will be collected for ~10 years in individuals who provide consent, conditional on future financial support (figure 1).

### Inclusion and exclusion

#### Inclusion criteria

- A diagnosis of acute ischaemic stroke or ICH, defined as rapidly developing clinical signs of focal (or global) cerebral dysfunction lasting more than 24 hours or leading to death, with no apparent cause other than vascular origin. The stroke diagnosis was made by specialist stroke physicians or neurologists, and in challenging cases, a panel of SIGNAL stroke physicians, neurologists and neuroradiologists reached a consensus on the final stroke diagnosis by consensus following review of presenting symptoms and signs supplemented by diagnostic brain MRI or CT and other clinically relevant investigations.
- Age 18 years and over.
- Resident within an NCL post code with routine follow-up planned in this stroke clinical pathway.

#### Exclusion criteria

- Individuals who had transient ischaemic attack (TIA), subarachnoid haemorrhage (SAH) or stroke mimics as the final diagnosis.
- Individuals under the age of 18 years old.

### Patient and public involvement

Ethnically diverse individuals with lived experience of stroke, along with caregivers, were actively involved in the design, methodological development and dissemination planning of the SIGNAL register. Engagement took place through a series of structured face-to-face and online meetings held during both the development and implementation phases of the registry. These consultations aimed to identify unmet needs after stroke, explore barriers to accessing care and prioritise under-studied areas in post-stroke recovery. Open discussions highlighted the importance of investigating non-motor outcomes, including anxiety, depression, fatigue, sleep disturbances, pain, social participation, bowel and bladder dysfunction, communication difficulties, mood problems and ADLs. In response, the SIGNAL register was designed to capture data on these outcomes, to better understand their burden and identify opportunities to improve access to care and support. All meetings were formally recorded, and feedback from individuals continued to directly inform the scope and priorities of the register.

### Data collected on admission

All variables collected at baseline are shown in table 1. Briefly, the first variable included stroke aetiology classified by a team of consultant stroke physicians, clinical fellows and a neuroradiologist from the SIGNAL team. Ischaemic stroke aetiology was classified according to the TOAST (Trial of Org 10 172 in Acute Stroke Treatment) criteria. ICH aetiology was determined using a modified version of the Causal Classification System for Intracerebral Haemorrhage Subtypes classification.^8^

**Table 1:** List of baseline variables collected in SIGNAL

Variables	Baseline	6 months (FU1)	10 years (FU2)
Age	x	–	–
Sex	x	–	–
Ethnic origin	x	–	–
Stroke type	x	–	–
Previous stroke/TIA	x	–	–
Hypertension	x	–	–
Dementia	x	–	–
Congestive heart failure	x	–	–
Diabetes mellitus	x	–	–
AF	x	–	–
Smoking	x	–	–
Admission NIHSS	x	–	–
Premorbid mRS	x	–	–
Discharge mRS	x	–	–
Thrombectomy	x	–	–
Thrombolysis	x	–	–
Antiplatelet on admission	x	–	–
Anticoagulant on admission	x	–	–
Antihypertensive on admission	x	–	–
Statin	X	–	–
CT scan on admission	x	–	–
CTA on admission	x	–	–
MRI on admission	x	–	–
MRA on admission	x	–	–
Home with ESD	x	–	–
ASU	x	–	–
Nursing/care home	x	–	–
Length of stroke unit stay	x	–	–

Table showing baseline variables.

AF, atrial fibrillation; ASU, acute stroke unit; CTA, CT angiography; ESD, early supported discharge; FU, follow-up; MRA, magnetic resonance angiography; NIHSS, National Institutes of Health Stroke Scale; SIGNAL, Stroke Investigation Group in North and Central London; TIA, transient ischaemic attack.

Data were extracted from individuals’ electronic health records (*Epic*, Epic Systems Corporation, Wisconsin, USA), including baseline sociodemographic and clinical factors (such as age, sex, onset time, medical history and medication history), admission diagnosis, stroke severity, length of stay and clinical outcomes, including the National Institutes of Health Stroke Scale (NIHSS) score and pre-stroke modified Rankin Scale (mRS).

The baseline data for individuals included in the registry were collected by trained clinical researchers from the SIGNAL team using a standardised data collection protocol and case report form developed by the SIGNAL Steering Committee. Prior to including eligible individuals in SIGNAL, both registry investigators and clinical research staff received Good Clinical Practice (GCP) training.

### Data collected at hospital discharge

The variables collected at discharge are also shown in table 1. In summary, at hospital discharge from UCLH Comprehensive Stroke Unit, a team of SIGNAL clinical researchers extracted data from the individuals’ discharge medication list, admission to brain imaging duration in days, discharge location (including acute stroke unit, home with early supported discharge (ESD), care home or home without ESD support) and mRS score on discharge. The contact information of individuals with a history of stroke and their next of kin, recorded in the electronic health records, was also verified at this stage.

### Brain imaging data

We collected the brain imaging data on admission to UCLH Comprehensive Stroke Unit, either or both via CT or MRI, including advanced sequences able to demonstrate intracranial vascular stenosis or occlusion, acute infarcts, macrohaemorrhages and microhaemorrhages, lacunes, white matter hyperintensities and perivascular spaces (allowing phenotyping of cerebral small vessel disease) in addition to structural anatomy.

The imaging protocol included: brain MRI (T1-weighted, T2-weighted, fluid-attenuated inversion recovery T2-weighted, diffusion-weighted imaging, susceptibility-weighted imaging and in a subset of individuals, diffusion tensor imaging with 6 directions); for those with ICH; and vascular assessment of intracranial and extracranial arteries using time-of-flight magnetic resonance angiography (MRA) or CT angiography (CTA), and extracranial arteries by carotid doppler ultrasound. In diagnostically challenging cases, contrast-enhanced magnetic resonance angiography (CE-MRA) or intra-arterial digital subtraction angiography was performed. The latter was also used when there was a high clinical suspicion of a macrovascular cause of ICH, or when endovascular treatments such as mechanical thrombectomy were indicated.

### 6-month follow-up data

The follow-up outcome measures are shown in table 2. In detail, the SIGNAL registry has two follow-up phases. These include the following: first, a 6-month follow-up of motor and non-motor outcomes for individuals admitted to the Comprehensive Stroke Unit between 1 January 2017 and 13 January 2020; and second, the planned long-term follow-up at ~10 years.

**Table 2:** List of follow-up variables collected in SIGNAL

Variables	Baseline	6 months (FU1)	10 years (FU2)
PROMIS-29			
Physical function	–	x	x
Anxiety	–	x	x
Depression	–	x	x
Fatigue	–	x	x
Sleep disturbance	–	x	x
Social roles and activities	–	x	x
Pain interference	–	x	x
Pain intensity	–	x	x
SIS-59			
Strength	–	x	x
Hand function	–	x	x
ADL/IADL	–	x	x
Mobility	–	x	x
Communication	–	x	x
Emotion	–	x	x
Memory and thinking	–	x	x
Social participation	–	x	x
Barthel Index			
Bowel	–	x	x
Bladder	–	x	x
Grooming	–	x	x
Toilet use	–	x	x
Feeding	–	x	x
Transfer	–	x	x
Mobility	–	x	x
Dressing	–	x	x
Stairs	–	x	x
Bathing	–	x	x
MOCA	–	x	–
FMA	–	x	–
TUG	–	x	–
ART	–	x	–
SPD	–	x	–

Table showing baseline variables.

ADL, Activities of Daily Living; ART, Aphasia Rapid Test; FMA, Fugl-Meyer Assessment of Motor Recovery; FU, follow-up; IADL, Instrumental Activities of Daily Living; MoCA, Montreal Cognitive Assessment; mRS, modified Rankin Scale; PROMIS-29, The Patient-Reported Outcomes Measurement and Information System-29; SIGNAL, Stroke Investigation Group in North and Central London; SIS-59, Stroke Impact Scale-59; SPD, Spoken Picture Description; TUG, Timed-Up-and-Go.

During the standardised 6-month stroke follow-up, multiple clinically approved follow-up methods were utilised to ensure person-centredness and equity of access to post-stroke support. These included face-to-face follow-ups at the UCLH outpatient stroke clinic or the individual’s residence, as well as telephone and postal follow-ups for those unable to attend the clinic. Individuals had the option of having a family member, friend or carer accompany them to the outpatient clinic or at their residential address to assist with completing the follow-up questionnaires. All data from individuals assessed at the outpatient clinic visits, telephone interviews, face-to-face follow-ups at individuals’ residential address and postal follow-ups were documented in the SIGNAL database. At each follow-up point, an individual was considered lost to follow-up if they failed to attend the outpatient clinic, could not be reached after three telephone contact attempts, or had not returned follow-up packs.

The comprehensive battery of follow-up measures used at 6-month follow-up included: mRS to capture functional status, and Questionnaire for Verifying Stroke-Free Status (QVSFS) to assess stroke recurrence; the Patient-Reported Outcomes Measurement and Information System-29 (PROMIS-29); Stroke Impact Scale-59 version 3.0 (SIS-59); the Hospital Anxiety and Depression scale (HADs); and Barthel Index score. These scores were used to comprehensively capture multi-domain non-motor outcomes (anxiety, depression, fatigue, sleep disturbance, social participation, pain, bowel dysfunction, bladder dysfunction, mood problems, communication problems, ADL (ADL), memory and thinking problems).

In 2018, additional outcomes were added to the SIGNAL outcome assessment battery. These included the Montreal Cognitive Assessment (MoCA) for cognitive screening; the Timed-Up-and-Go (TUG) for balance; the Spoken Picture Description (SPD) for communication; and the Fugl-Meyer Assessment of Motor Recovery (FMA) for a performance-based limb impairment index.

The longer-term follow-up, approved as a research study with consent rather than a clinical registry, at ~10 years post-stroke will repeat the 6-month battery of outcome measures, with additional outcomes that are important for life after stroke. This follow-up study began in September 2024 for the young stroke cohort (<55 years old) within the SIGNAL registry, and we are currently seeking funding to extend follow-up to the full registry cohort. These added outcome measures will include return to work, financial-resource strain, relationship status and sexual function. The SIGNAL registry is overseen by a local UCLH BRC-funded Steering Committee that includes stroke physicians and other researchers.

### Data storage

Initially, SIGNAL data were collected using a paper case report form. This information was subsequently entered into a fully anonymised electronic data capture system secured within the UCLH firewall. At the end of each working day, clinical research practitioners entered the baseline demographic and clinical data into the electronic database, authenticated by their electronic signatures (unique username and password).

All data entries were rigorously checked for completeness, correct diagnosis and value ranges against the hospital records by the SIGNAL registry team. Any amendments to the baseline dataset were recorded with an electronic audit trail that included the date of modification. Independent data monitoring was conducted throughout the registry period by two GCP-trained clinical researchers to ensure data integrity.

### Statistical analysis

The baseline sociodemographic and clinical data were directly captured from electronic health records. Each project conducted within SIGNAL will use appropriate statistical analyses to test specific hypotheses. Typically, proportions will be used to describe categorical data and means with SD or medians with IQR will be used to describe continuous data. The t-test or Mann-Whitney test will be used to compare groups of continuous data, while Pearson’s χ^2^ test or Fisher’s exact test will be used to compare categorical data. We will assess the strength of any identified associations and adjust for potential confounding using regression models. We will use logistic regression models for dichotomous outcomes and linear regression models for continuous outcomes. Detailed descriptions of these methods will be provided in each project publication derived from SIGNAL data.

For this protocol, since the baseline registration and the standardised 6-month follow-up are finished, we performed descriptive statistical analyses to report baseline characteristics of individuals included in the SIGNAL registry and the proportion of people with follow-up. We present the number of individuals (N) and proportions (%) for categorical variables, and, for continuous variables, means with SD, or medians with IQR. All statistical analyses were performed on STATA V.16.2.

## Findings to date

### Sociodemographic characteristics

**Figure 2:**
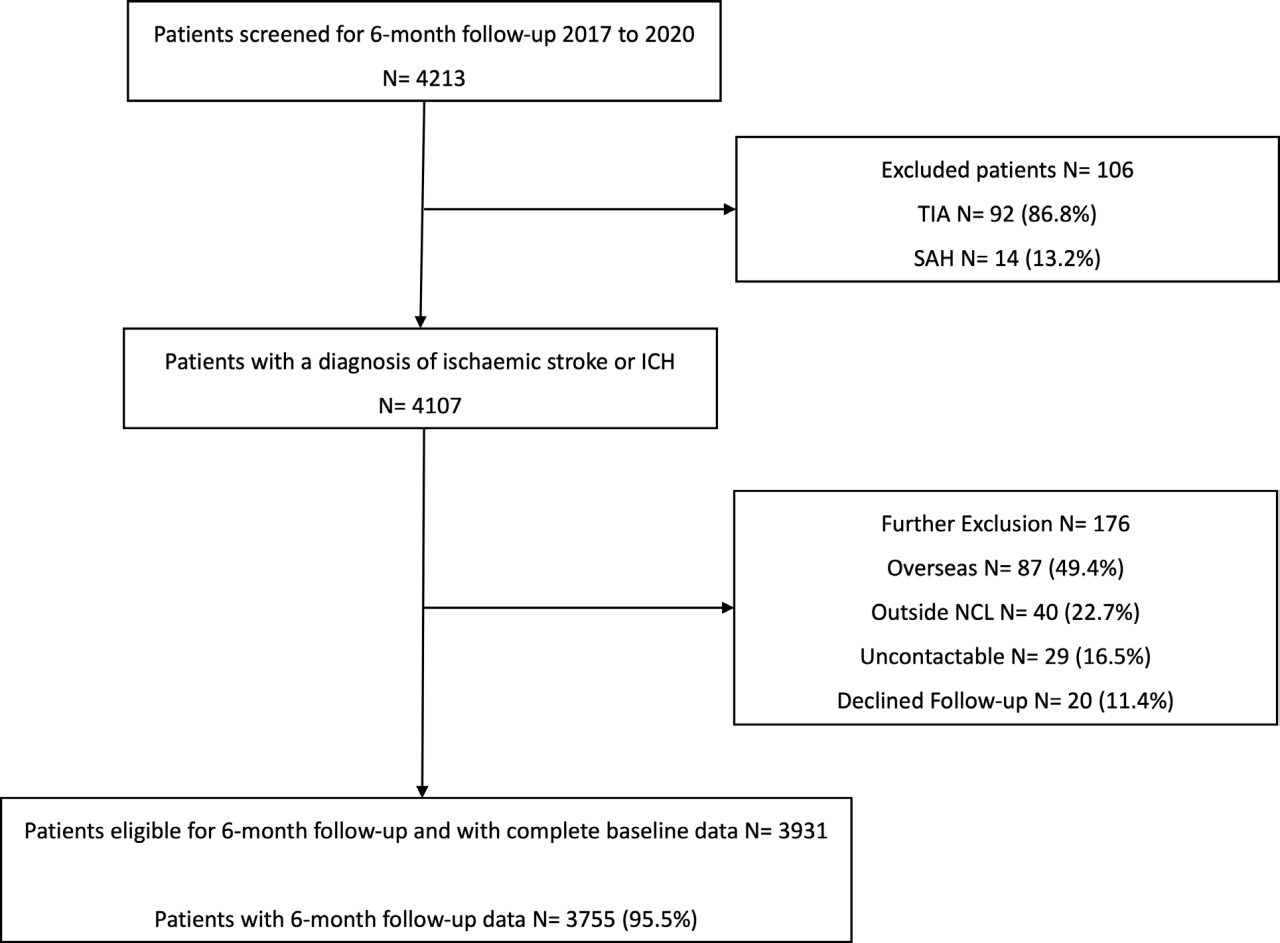
Flow diagram of SIGNAL registry. *Flowchart of patient inclusion in SIGNAL from admission to 6-month follow-up. ICH, intracerebral haemorrhage; NCL, North Central London; SAH, subarachnoid haemorrhage; SIGNAL, Stroke Investigation Group in North and Central London; TIA, transient ischaemic attack.

Between January 2017 and January 2020, 4213 individuals were admitted to the comprehensive stroke services at UCLH. Of these, 282 were excluded from the SIGNAL registry: 92 had a diagnosis of TIA, 14 had subarachnoid haemorrhage (SAH) and 176 did not meet other eligibility criteria. See figure 2 for a detailed overview of inclusion and exclusion criteria. A total of 3931 individuals were included in the registry, of whom 3755 (95.6%) had complete 6 month follow-up data.

Table 3 provides a detailed overview of the baseline variables and individual data points captured within the SIGNAL database. The cohort had a median age of 72.1 years (SD±24.1), 1806 (45.9%) were female, 2365 (60%) were ethnically white, 649 (16.5%) black and 511 (13%) Asian.

**Table 3:** Baseline data description

Variables	Number of participants N=3931
Age (years), mean SD	72.1±15.1
Sex, female, N (%)	1806 (45.9%)
Ethnic origin, N (%)	
White	2365 (60.2%)
Black	649 (16.5%)
Asian	511 (13%)
Other	216 (5.5%)
Not known	190 (4.8%)
Stroke type, N (%)	
Ischaemic stroke	3371 (85.8%)
Intracerebral haemorrhage	560 (14.2%)
Medical history, N (%)	
History of stroke/TIA	703 (17.9%)
Hypertension	2506 (63.7%)
AF	956 (24.3%)
Heart failure (3929 complete)	183 (4.7%)
Diabetes mellitus	1034 (26.3%)
Acute treatments	
Thrombolysis (3930 complete)	616 (18.1%)
Thrombectomy	294 (7.5%)
Clinical features, N (%)	
Admission NIHSS 0–3	1688 (43.0%)
Admission NIHSS ≥4	2240 (57.0%)
Premorbid mRS	3931 (100%)
Discharge mRS	3931 (100%)
Discharge mRS, median (IQR)	
Brain imaging, N (%)	
CT (at UCLH)	3596 (95.6%)
CTA (at UCLH)	2231 (62%)
MRI (at UCLH)	2234 (57%)
MRA (at UCLH)	368 (16.5%)

Table showing baseline characteristics and completeness of clinical and brain imaging data.

AF, atrial fibrillation; CTA, CT angiography; ESD, early supported discharge; MRA, magnetic resonance angiography; mRS, modified Rankin Scale; NIHSS, National Institute of Health Stroke Severity Scale; TIA, transient ischaemic attack; UCLH, University College London Hospitals.

### Clinical characteristics

A total of 3371 individuals (85.8%) were diagnosed with acute ischaemic stroke, while 560 (14.2%) diagnosed with ICH. On admission, 2240 (57.0%) individuals had an NIHSS score >4, indicating moderate stroke severity. The median functional outcome at discharge, measured by the mRS, was 3 (IQR 1–4), indicating moderate disability. A total of 703 (17.9%) individuals had a history of stroke or TIA, 2506 (63.7%) hypertension, 956 (24.3%) atrial fibrillation, 183 (4.7%) heart failure and 1034 (26.3%) diabetes mellitus. On admission, 3596 (95.6%) had CT brain imaging and 2234 (57.0%) had MRI.

### 6-month follow-up and outcomes

Table 4 provides a detailed overview of the 6-month variables and the proportions of follow-up data points captured. Briefly, at 6 months, functional outcome data were available for 3755 (95.6%) individuals, with a median mRS score of 1 (IQR 0–3). Stroke recurrence data were captured for 2144 (54.5%) individuals. Multi-domain non-motor outcome data were available for 3080 (92.3%) individuals, for which results have been published.^9^ In brief, the most prevalent adverse non-motor outcomes were: fatigue 1756 (57%); reduced social participation 1694 (55%); sleep disturbance 1663 (54%); and constipation 355 (44%). Less common adverse non-motor outcomes were: memory and thinking 708 (23%); ADL 860 (28%); anxiety 860 (28%); and communication problems 892 (29%).^9^ The factors most strongly associated with adverse non-motor outcomes were stroke due to ICH, admission NIHSS score ≥5, history of stroke or TIA and a history of cardiovascular disease such as hypertension.^9^

Further 6-month follow-up data were collected between 2018 and 2020 from 1727 individuals as part of routine data collection and remain unpublished. These included measures of cognition as measured with MoCA 1604 (93%), balance with TUG 1429 (83%), communication problems with SPD 872 (50.4%) and performance-based limb impairments with FMA 1506 (87%).

**Table 4:** 6-month follow-up rate

Variables	Number of participants N=3931
Primary follow-up outcomes capture rate, N (%)	
6-month mRS	3755 (95.6%)*
6-month mRS, median (IQR)	1 (0–3)
Stroke recurrence data captured with QVSFS	2144 (54.5%)
Data captured for patient-reported health outcomes N (%), N=3338	
Patients alive and eligible for 6-month follow-up	
PROMIS-29	3080 (92.3%)
Stroke Impact Scale version 3.0 (SIS-59)	3065 (92%)
Barthel Index	3079 (92.2%)
HADs	2744 (82%)
Follow-up methods, N (%)	3080
Face to face	1829 (59.4%)
Telephone	732 (23.8%)
Postal	392 (12.7%)
Proxy responder	127 (4.1%)
Detailed 6 months data (patients alive at 6-month N=1727) collection commenced 1 September 2018	
MOCA	1604 (93%)
FMA	1506 (87%)
TUG	1429 (83%)
ART	796 (46%)
SPD	872 (50.4%)
Added 5–10 years of outcomes commenced in September 2024	
Return to work and financial-resource strain	–
Relationship status	–
Sexual function	–

Table showing follow-up rate at 6-month post-stroke.

There is a small proportion of missing data in the NIHSS and thrombolysis variables.

* Limited number of brain CT and MRI were conducted at a transferring centre.

ADL, Activities of Daily Living; ART, Aphasia Rapid Test; FMA, Fugl-Meyer Assessment of Motor Recovery; HADs, Hospital Anxiety and Depression scale; IADL, Instrumental Activities of Daily Living; MoCA, Montreal Cognitive Assessment; mRS, modified Rankin Scale; PROMIS-29, The Patient-Reported Outcomes Measurement and Information System-29; QVSFS, Questionnaire for Verifying Stroke-Free Status; SIS-59, Stroke Impact Scale-59; SPD, Spoken Picture Description; TUG, Timed-Up-and-Go.

### Strengths and limitations

The strengths of the SIGNAL registry are: its large sample size; the detail and completeness of clinical and brain imaging phenotypes; the large range of clinician and patient-reported outcomes; and the lack of selection bias, making results directly applicable to routine clinical practice in similar healthcare settings. SIGNAL holds detailed brain imaging data for 3596 (95.6%) individuals with stroke. By comparison, the South London Stroke Registry has 6052 participants, of whom 25% have available brain imaging.^2^ The higher rate of MRI and dedicated vascular imaging will enable more precise classification, and we should be able to investigate novel associations of stroke subtypes and neuroimaging with outcomes. In line with our study, the Oxford Vascular Study achieved 96% brain-imaging capture; however, its predominantly white (94%) and less-deprived population limits generalisability, to populations with different ethnic and socioeconomic characteristics.^3^ Moreover, its narrower non-motor outcome assessment underscores the need for more inclusive cohorts with broader, patient-centred measures.

A novel feature of our registry is the number of patient-reported outcomes collected across multiple non-motor outcome domains with very high data completeness. We are not aware of UK-based stroke studies with a higher rate of 6-month patient-reported multi-domain non-motor outcomes that define the full contemporary natural history of life after stroke. These comprehensive outcomes are combined with detailed neuroimaging including high rates of non-invasive angiography and MRI.^9^ Stringent eligibility criteria used by randomised controlled trials in stroke to date mean that the accepted gold standard of evidence to guide clinical practice tends to select against those who are older or have more baseline frailty or comorbidity.^10^ Observational research from unselected cohorts, such as SIGNAL, is potentially important to guide stroke care in these high-risk groups where randomised evidence is lacking.

We also acknowledge limitations. The main limitation of the SIGNAL registry is that assessment of the effects of interventions could be influenced by residual confounding of results by indication and baseline imbalances, particularly age and pre-morbid functional status. This is mitigated to an extent by the unselected population (reducing bias) and planned adjusted analyses. We also acknowledge that relying on electronic health records to capture baseline cardiovascular risk factors may introduce misclassification or detection bias, particularly for conditions such as AF or hypertension around the time of stroke. This limitation was addressed by defining cardiovascular risk factors using data obtained during the index stroke admission. A further limitation is the lack of consent to access primary care records for earlier medical history. Where feasible, however, we qualitatively captured key pre-stroke non-motor outcomes such as memory concerns, anxiety and depression using patient self-report and information from carers or next of kin. In addition, the multi-borough design centred on a single metropolitan stroke service may limit generalisability to other settings, particularly rural or non-metropolitan regions. Finally, the capture of 6-month stroke-recurrence data was incomplete, primarily due to staffing strain and the delayed introduction of systematic follow-up procedures. Although recurrence data were available for only 54.5% of the cohort, this level of follow-up is comparable to several large, published stroke registries and is a recognised challenge in real-world registry-based research.

## Summary and conclusions

SIGNAL has systematically gathered comprehensive data on clinical, brain imaging, motor, language, cognitive and non-motor outcomes in a large, ethnically diverse and unselected stroke population. The precise subtyping of strokes, combined with detailed outcome measures, will generate new hypotheses for future stroke research. By providing robust data on the mechanisms of acute ischaemic stroke and ICH through brain imaging and systematic clinical data collection, SIGNAL will facilitate the stratification of outcomes by stroke type and improve the identification of risk factors for adverse outcomes. Additionally, SIGNAL plans to collect longer-term detailed follow-up data at ~10 years after stroke, pending future funding.

## Data Availability

Data are available upon reasonable request.
