# Supplementary material for: Stroke Investigation Group in North and Central London (SIGNAL): cohort profile of a prospective large-scale comprehensive stroke registry
Source: BMJ Open. 2026 Jan 30;16(1):e110772. doi: 10.1136/bmjopen-2025-110772 (PMC12863321; doi:10.1136/bmjopen-2025-110772)
Supplement: Supplementary file 1 [file bmjopen-2025-110772.reviewer_comments.pdf]

PEER REVIEW HISTORY

BMJ Open publishes all reviews undertaken for accepted manuscripts. Reviewers are asked to complete a checklist review form and are provided with free text boxes to elaborate on their assessment. These free text comments are reproduced below.

ARTICLE DETAILS

Title (Provisional)

Stroke Investigation Group in North And Central London (SIGNAL): cohort profile of a prospective large-scale comprehensive stroke registry

Authors

Ozkan, Hatice; Ambler, Gareth; Nash, Philip S; Browning, Simone; Mussa, Raafiah; Leff, Alex; Jäger, Hans R.; Nachev, Parashkev; Perry, Richard; Chan, Edgar; Simister, Robert; Werring, David J; SIGNAL, collaborators

---

VERSION 1 - REVIEW

**Reviewer**	**1**
**Name**	**Sung, Sheng-Feng**
**Affiliation**	**Ditmanson Medical Foundation Chia-Yi Christian Hospital, Division of Neurology, Department of Internal Medicine**
**Date**	**20-Oct-2025**
**COI**	**None**

This manuscript describes a hospital-based stroke registry in North Central London that recruits an ethnically diverse population. The registry collects demographic, clinical, and brain imaging data. A key feature is its collection of six-month functional and multi-domain non-motor outcomes, including mood, cognition, fatigue, sleep, pain, language, social participation, and bowel and bladder function. The registry aims to collect long-term follow-up data for up to 10 years, though this is temporarily paused due to funding constraints. While the manuscript is clear and straightforward, several aspects require further elaboration:

Major comments:

• Many similar stroke registries exist around the world, including in the UK. A more detailed comparison of the similarities and differences between this registry and existing ones would help justify the establishment of a new registry.

• Since the objective of this manuscript is to introduce the registry, it would be helpful to define important data elements, such as risk factors, perhaps in supplementary material. For

example, readers would want to know whether the AF variable indicates known AF or includes newly diagnosed AF after stroke admission.

• The registry collects brain imaging data, but Table 1 does not specify which data elements are captured for CT, CTA, MRI, and MRA.

• During the collection of admission details, did clinical researchers conduct face-to-face interviews with patients, or did they extract data solely from electronic health records? Please clarify. If only data extraction was performed, how was missing data handled?

• Since patients were followed up through multiple routes, including outpatient clinic assessments, telephone interviews, face-to-face home visits, and postal surveys, what proportion did each route represent? How did the registry maintain data quality across these diverse sources? Even though all data entries were checked by the registry team, how were questionable data traced back to their source?

• The authors highlight the lack of selection bias as a key strength of this registry. However, single-center registries may not represent the broader stroke population, since patient demographics, referral patterns, and clinical practices can vary significantly between institutions. Please discuss how North Central London's ethnically diverse catchment area might mitigate or worsen this concern. Additionally, are there plans to validate findings against other regional or national stroke registries?

**Reviewer**	**2**
**Name**	**Chan, Bernard**
**Affiliation**	**National University Health System, Medicine**
**Date**	**29-Oct-2025**
**COI**	**None**

The authors reported the protocol of a prospective hospital-based stroke registry, and its preliminary 6-month follow up data, with the distinction of including a number of non-motor outcomes. I have a few minor queries:

1. The authors should explain why the first follow up was selected at 6 months. Most studies on ischaemic stroke outcomes had the primary follow up at 3 months, especially for mild strokes. A late follow up may also decrease follow up rates and some of the non-motor outcomes might have occurred and remitted by 6 months. Therefore the authors should also discuss whether outcomes that occurred earlier could be captured (e.g. review of case notes/prescription lists, reports by GP).

2. In Table 1, concerning the items antiplatelet on admission, anticoagulation on admission, antihypertensive on admission and statin, could the authors clarify whether they refer to pre-existent treatments before stroke onset?

3. In the section 6-month follow-up and outcomes, it was reported "Stroke recurrence data were captured for 2144 (54.5%)" and not clarified later whether the remaining patients' data were available. This proportion appears too low for a stroke registry.

VERSION 1 - AUTHOR RESPONSE

Reviewer: 1
Dr. Sheng-Feng Sung, Ditmanson Medical Foundation Chia-Yi Christian Hospital

Comments to the Author:
This manuscript describes a hospital-based stroke registry in North Central London that recruits an ethnically diverse population. The registry collects demographic, clinical, and brain imaging data. A key feature is its collection of six-month functional and multi-domain non-motor outcomes, including mood, cognition, fatigue, sleep, pain, language, social participation, and bowel and bladder function. The registry aims to collect long-term follow-up data for up to 10 years, though this is temporarily paused due to funding constraints. While the manuscript is clear and straightforward, several aspects require further elaboration:
Major comments:

1. Many similar stroke registries exist around the world, including in the UK. A more detailed comparison of the similarities and differences between this registry and existing ones would help justify the establishment of a new registry.

    - We thank the reviewer for their well highlighted comment, we have addressed the comment in detail in the introduction, please see lines 119 to 135.

    - We have also addressed this valuable comment in the discussion section lines 401 to 407.

2. Since the objective of this manuscript is to introduce the registry, it would be helpful to define important data elements, such as risk factors, perhaps in supplementary material. For example, readers would want to know whether the AF variable indicates known AF or includes newly diagnosed AF after stroke admission.

    - We thank the reviewer for this valuable comment. Baseline data were extracted directly from clinical records, and we were unable to stratify by differences in the timing of AF diagnosis. This limitation is now acknowledged in the manuscript (please see Discussion, lines 425–428).

3. The registry collects brain imaging data, but Table 1 does not specify which data elements are captured for CT, CTA, MRI, and MRA.

    - We thank the reviewer for their valuable comment. A detailed description of the brain markers captured in our study is provided in the Results section (lines 262–279), and the proportions of CTA and MRA derived from CT and MRI imaging have now been added to the updated Table 1.

4. During the collection of admission details, did clinical researchers conduct face-to-face interviews with patients, or did they extract data solely from electronic health records? Please clarify. If only data extraction was performed, how was missing data handled?

- We thank the reviewer for their insightful comment. We confirm that admission details were directly extracted from clinical health records, and any missing data were carefully checked during the 6-month follow-up. The small proportion of missing data in the NIHSS was addressed on a case-by-case basis, as is typical when analysing NIHSS data. For the thrombolysis variable, missing data were handled under the assumption of missing completely at random (MCAR), given that the missingness is minimal (<5%).

5. Since patients were followed up through multiple routes, including outpatient clinic assessments, telephone interviews, face-to-face home visits, and postal surveys, what proportion did each route represent?

- We thank the reviewer for their thoughtful comment. We confirm that patients were followed up through multiple routes, including outpatient clinic assessments, telephone interviews, face-to-face home visits, and postal surveys. The proportion of patients (N= 3080) using each route for follow-up was as follows: Face-to-face home visits: 1829 patients (59.4%); Telephone interviews: 732 patients (23.8%); Postal surveys: 392 patients (12.7%); Proxy responders: 127 patients (4.1%). Please updated see table 1.

- We note that all outcome measures used in our study are widely validated for proxy use. To ensure the inclusion of all eligible patients, we implemented supplementary supportive measures for follow-up. Specifically, for patients with moderate to severe communication issues, cognitive impairment, language barriers (e.g., non-English speakers), or severe functional disability (mRS 4–5) who could not travel to outpatient clinics, we offered alternative follow-up options such as extended follow-up time, telephone assessments, postal questionnaires, or translated outcome measures with the assistance of a proxy responder. This approach ensured broad participation and minimized follow-up bias.

6. How did the registry maintain data quality across these diverse sources? Even though all data entries were checked by the registry team, how were questionable data traced back to their source?

- We thank the reviewer for their important question. To ensure data quality across the diverse follow-up sources, we implemented standardised data collection protocols, with all entries carefully reviewed by the registry team. Widely validated outcome questionnaires were used across all assessment methods, including face-to-face visits, telephone interviews, postal surveys, and proxy assessments, ensuring consistency and reliability across all follow-up routes. For example, anxiety and depression outcomes were reviewed with a neuropsychology consultant for patients with cognitive impairment, while patients with severe functional disability or communication difficulties were assisted by proxy

responders to ensure accurate reporting. In cases of questionable data, discrepancies were traced back to their source through follow-up contact (e.g., telephone calls, home visits, or clarification with healthcare providers). Regular audits and cross-validation of data further ensured consistency across different sources, while ongoing training for registry staff minimised errors and improved data accuracy.

7. The authors highlight the lack of selection bias as a key strength of this registry. However, single-center registries may not represent the broader stroke population, since patient demographics, referral patterns, and clinical practices can vary significantly between institutions. Please discuss how North Central London's ethnically diverse catchment area might mitigate or worsen this concern. Additionally, are there plans to validate findings against other regional or national stroke registries?

   - We thank the reviewer for their insightful comment. We acknowledge that single-centre registries can introduce limitations related to generalisability, particularly given potential variations in patient demographics, referral patterns, and clinical practices across different institutions. However, North Central London's ethnically diverse catchment area is a key strength of our registry, as it helps mitigate concerns regarding selection bias. The population served by our centre includes individuals from a wide range of ethnic backgrounds, which increases the representativeness of the data and provides valuable insights into stroke care and outcomes across diverse groups. This diversity helps ensure that the registry reflects the broader stroke population in the UK, which is increasingly multicultural.

   - The **UCLH Comprehensive Stroke Service** offers comprehensive and high-quality care, including access to specialist stroke units, advanced neuroimaging facilities, multidisciplinary care teams, and a robust rehabilitation programme, all of which are key strengths that contribute to the high standard of data collected in our registry.

   - While we believe that the demographic breadth of our catchment area reduces the potential for selection bias, we recognise that variations in clinical practices across different healthcare settings could influence outcomes. To address this, we are currently collaborating with the **South London Stroke Registry** to validate some of our findings, enabling us to cross-check our data against a complementary regional registry. This collaboration will help ensure the robustness and generalisability of our results by allowing for cross-comparison with a different but similarly diverse population. Furthermore, we are exploring collaborations with other regional and national stroke registries to assess the consistency of our findings across diverse healthcare settings and to validate our conclusions on a broader scale.

Reviewer: 2
Dr. Bernard Chan, National University Health System
Comments to the Author:

The authors reported the protocol of a prospective hospital-based stroke registry, and its preliminary 6-month follow up data, with the distinction of including several non-motor outcomes. I have a few minor queries:

1. The authors should explain why the first follow up was selected at 6 months. Most studies on ischaemic stroke outcomes had the primary follow up at 3 months, especially for mild strokes. A late follow up may also decrease follow up rates and some of the non-motor outcomes might have occurred and remitted by 6 months. Therefore, the authors should also discuss whether outcomes that occurred earlier could be captured (e.g. review of case notes/prescription lists, reports by GP).

- We thank the reviewer for their valuable comment. The 6-month follow-up is standard for the first clinical follow-up, as our registry is integrated into the clinical services provided at UCLH. This timing aligns with the typical transition from acute stroke units (ASU) to established community care, with patients often discharged to either residential or community settings. While 3-month follow-up is commonly used in research studies to assess acute treatments, trials, and early functional outcomes, the 6-month time point is more appropriate for capturing longer-term clinical recovery. This includes not only physical rehabilitation but also communication rehabilitation and a more comprehensive range of non-motor outcomes. At this point, patients are generally under the ongoing care of neurology services, and any emerging non-motor issues, such as depression or anxiety, can be more effectively identified, managed, or referred to appropriate services.

- Our registry has achieved a follow-up capture rate of over 90%, which has enabled us to include not only patient outcomes but also the perspectives of carers and next of kin (NOK), adding depth to our data on recovery. Moreover, due to the clinical approval of our registry, we do not have consent to access GP records to capture earlier data. However, where possible, we have qualitatively documented patients' pre-stroke history of non-motor outcomes, such as memory problems, anxiety and depression, based on patient self-reports or information provided by carers and next of kin. We have also acknowledged this in the limitations section; please see Discussion, lines 438–440.

2. In Table 1, concerning the items antiplatelet on admission, anticoagulation on admission, antihypertensive on admission and statin, could the authors clarify whether they refer to pre-existent treatments before stroke onset?

- We thank the reviewer for this important point. The baseline admission medication list includes medications taken prior to admission as well as those initiated at the time of admission.

3. In the section 6-month follow-up and outcomes, it was reported "Stroke recurrence data were captured for 2144 (54.5%)" and not clarified later whether the remaining patients' data were available. This proportion appears too low for a stroke registry.

- We thank the reviewer for highlighting this important point. We agree that the proportion of patients with documented 6-month stroke-recurrence data appears low for a stroke registry and acknowledge the need for clarification. As noted,

recurrence outcomes were available for 2,144 patients (54.5%). For the remaining patients, 6-month follow-up data were unavailable mainly due to staffing strain, particularly during the early phase of registry implementation. In addition, systematic 6-month follow-up procedures were introduced only three months after the initiation of the registry, which further contributed to the lower follow-up rate.

- However, the overall cohort size remains large, and the proportion of recurrence data captured is comparable to several published stroke registries, such as the South London Stroke Register (N = 2,874), many of which report 6-month follow-up completeness in the range of approximately 50–65%, depending on available resources and follow-up mechanisms. We have now clarified this in the manuscript and incorporated this point into the Discussion section lines 443-448.

VERSION 2 - REVIEW

Reviewer 1

Name Sung, Sheng-Feng

Affiliation Ditmanson Medical Foundation Chia-Yi Christian Hospital, Division of Neurology, Department of Internal Medicine

Date 17-Dec-2025

COI

I want to express my gratitude to the authors. All of my concerns have been resolved in the revised manuscript.

Reviewer 2

Name Chan, Bernard

Affiliation National University Health System, Medicine

Date 24-Dec-2025

COI

I thank the authors for addressing the queries of the reviewers and making the amendments. I have no further questions or concerns.
